# Safety of Concentrated Bioshell Calcium Oxide Water Application for Surface and Skin Disinfections against Pathogenic Microbes

**DOI:** 10.3390/molecules25194502

**Published:** 2020-10-01

**Authors:** Masayuki Ishihara, Yuuki Hata, Sumiyo Hiruma, Tomohiro Takayama, Shingo Nakamura, Yoko Sato, Naoko Ando, Koichi Fukuda, Kaoru Murakami, Hidetaka Yokoe

**Affiliations:** 1 Division of Biomedical Engineering, National Defense Medical College Research Institute, 3-2 Namiki, Tokorozawa, Saitama 359-8513, Japan; hata@ndmc.ac.jp (Y.H.); iroihsh@gmail.com (S.H.); snaka@ndmc.ac.jp (S.N.); naoandokoro@gmail.com (N.A.); khf05707@nifty.com (K.F.); 2 Department of Oral and Maxillofacial Surgery, National Defense Medical College Hospital, 3-2 Namiki, Tokorozawa, Saitama 359-8513, Japan; taka01@ndmc.ac.jp (T.T.); murakami@ndmc.ac.jp (K.M.); yokoe@ndmc.ac.jp (H.Y.); 3 Division of Statistical Analysis, Research Support Center, Shizuoka General Hospital, 4-27-1 Kita-ando, Aoi-ku, Shizuoka 420-8527, Japan; sato.yoko.shiz@gmail.com

**Keywords:** bioshell calcium oxides, calcium carbonate, strong alkalinity, disinfectant, microbicidal activity, safety

## Abstract

Immediately post-production, commercially available bioshell calcium oxide (BiSCaO) water is colorless, transparent, and strongly alkaline (pH 12.8), and is known to possess deodorizing properties and broad microbicidal activity. However, BiSCaO Water may represent a serious safety risk to the living body, given the strong alkalinity. This study aimed to investigate the safety of BiSCaO Water for use as an antiseptic/disinfectant despite concerns regarding its high alkalinity. The change over time in pH of BiSCaO Water was measured during air contact (stirring BiSCaO Water in ambient air). When sprayed on metal, plastic, wood piece, paper, and skin surfaces, the pH of BiSCaO Water decreased rapidly, providing a white powder coating upon drying. Scanning electron microscopy images, energy dispersive X-ray elemental mapping, and X-ray diffractograms showed that the dried powder residues of BiSCaO Water were composed primarily of calcium carbonate. These results suggested that BiSCaO Water is a potent reagent that may overcome the obstacles of being strongly alkaline, making this material appropriate for use in disinfection against pathogenic microbes.

## 1. Introduction

The recent epidemic of coronavirus disease (COVID-19), caused by a newly discovered coronavirus SARS-CoV-2, is a worldwide crisis [1,2,3]. Some antiseptics/disinfectants, such as ethanol and NaClO, show significant activity with broad microbicidal and antiviral activities, notably, activity against SARS-CoV-2 results from disruption of the viral envelope. However, such antiseptics/disinfectants are cytotoxic to cellular and organic components [4,5,6] and require high concentrations for disinfection activity [4,5,6]. Furthermore, chlorine-derived disinfectants are ineffective in the presence of organic compounds and may generate compounds harmful to the environment and living tissue [7,8,9]. Therefore, novel disinfectants that can decrease the bacterial and viral bioburden without harmful side effects and environmental disruption are still desired for environmental hygiene and public health.

Recently, bioshell calcium oxide (BiSCaO) has gained considerable attention as an emerging disinfectant. BiSCaO is prepared from scallop shells, which are mainly composed of CaCO_3_, by heating above 800 °C to convert them to CaO. Intriguingly, this material has been demonstrated to exhibit broad antimicrobial activity against various pathogenic bacteria [10,11], avian influenza virus [12], heat-resistant bacterial spores [13], fungi [14], and biofilms [15,16,17]. Furthermore, BiSCaO can be used as an additive to prolong the shelf life of foods [10,13]. One of the mechanisms whereby BiSCaO has disinfection activity is likely based on a strong base produced by hydration of CaO with water to be Ca(OH)_2_. Meanwhile, other mechanisms appear to be involved in the disinfection activity of BiSCaO, given the fact that the disinfection activity of CaO towards both total viable bacterial cells (TC) and coliform bacteria (CF) is higher than that of Ca(OH)_2_ or NaOH solution at the same pH [18,19]. Significantly, at the same concentrations, BiSCaO has shown to exhibit greater disinfection activity than conventional disinfectants, including HOCl (pH 6.5), NaClO (pH 9.5), povidone-iodine, and chlorhexidine gluconate [18,19].

We have investigated potential application of BiSCaO for wound treatment. It is known that daily cleaning of wounds with HClO solution (pH 6.5) decreases the *Pseudomonas aeruginosa* bioburden of infected wounds in *db/db* diabetic mice, but it delays wound healing [9]. To overcome the negative effects of HClO on wound healing, strategies such as the use of chitin-nanofiber sheet-immobilized silver nanoparticles (CNFS/Ag NPs) along with less HClO treatments have been investigated [19,20,21,22,23]. In this context, BiSCaO can remarkably disinfect wounds while maintaining healing; when *P. aeruginosa*-infected wounds on hairless rats were treated with a BiSCaO suspension (0.2 wt.%, pH 12.3) [24] or a BiSCaO-containing ointment [25] once daily for 3 days with covering the wounds with CNFS, wound healing was enhanced with significant decrease in the *P. aeruginosa* bioburden [23].

Despite the superior disinfection activity, the poor water-solubility of BiSCaO under strongly alkaline conditions (pH > 12.3) has been an obstacle for practical application. For instance, spray nozzles tend to be plugged with precipitates produced from water suspensions containing a high concentration of BiSCaO [12,26]. The addition of phosphate compounds such as Na_2_HPO_4_ [27] or Na-polyPO_4_ (PP) [28] to the BiSCaO-containing water has shown to partially solve the problem of the precipitation. BiSCaO dispersion is a light white semi-transparent dispersion without precipitations [27]. And BiSCaO colloidal dispersion is composed of transparent layer in supernatant and white flocculation layer which rapidly formed as easy dispersible layer [28]. Deodorization and microbicidal activities of the resulting BiSCaO dispersions and colloidal dispersions were higher than those of BiSCaO suspension [27,28].

Alternatively, we recently reported a novel BiSCaO-based disinfectant termed BiSCaO Water, which could be prepatablered by repeatedly adding BiSCaO to chilled clean water and gently decanting the supernatant into a separate container. The resulting BiSCaO Water supernatant with a pH > 12.7 was revealed by in vitro assays to eliminate more than 99.9% of viruses such as Influenza A (H1N1), Feline calicivirus, and bacteria such as *Escherichia coli* strains NBRC 3972 and O-157:H7, *Pseudomonas aeruginosa*, *Salmonella*, and *Staphylococcus aureus* within 15 min [29]. This BiSCaO Water lacked large particles but contained small aggregates (about 400–800 nm in size) of nanoparticles (100–200 nm), and, thus, was transparent [29]. BiSCaO Water has higher deodorization and disinfection activities than do other microbicidal reagents such as ethanol, BiSCaO, BiSCa(OH)_2_ suspensions, povidone iodine, NaClO, BiSCaO dispersion, and BiSCaO colloidal dispersion [29]. However, BiSCaO Water represents a serious safety risk to the living body, given that the pH of BiSCaO Water exceeds 12.7. The present study aimed to investigate the safety of BiSCaO Water when employed as an antiseptic/disinfectant despite this reagent’s strong alkalinity.

## 2. Results

### 2.1. Scanning Electron Microscopy (SEM) Images of BiSCaO Water and X-ray Diffractogram of the Dried Powder

**Figure 1:**
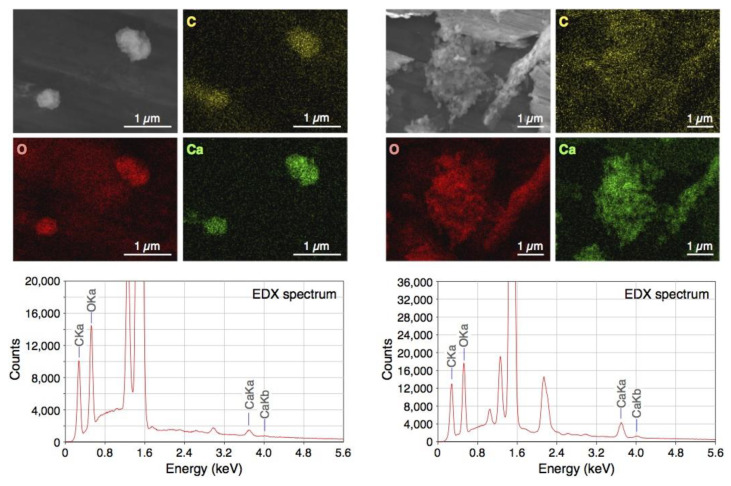
Scanning Electron Microscopy (SEM) and energy dispersive X-ray (EDX) elemental mapping images and EDX spectra of freeze-dried bioshell calcium oxide (BiSCaO) Water. Two different regions of the sample were analyzed (left and right). Yellow, red, and green pictures show the presence of carbon, oxygen, and calcium, respectively, by EDX elemental mapping.

**Figure 2:**
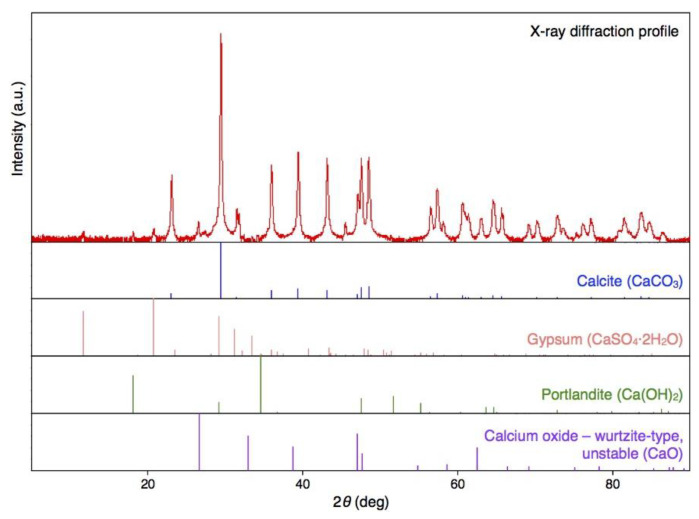
The CaCO_3_ content of the white powder obtained upon air-drying of BiSCaO Water. X-ray diffraction demonstrated that the dry powder obtained from BiSCaO Water consisted primarily of CaCO_3_.

BiSCaO Water was freeze-dried for SEM observation and energy dispersive X-ray (EDX) elemental mapping of particles contained. The images and EDX spectra are shown in Figure 1. The EDX elemental mapping showed that the particles contained in BiSCaO Water were composed of oxygen, carbon, and calcium. The dried white powder was insoluble in water, and the pH of the supernatant of a suspension of this powder was below 10. These results suggested that the observed particles were generated by an interaction between Ca^2+^ ions in BiSCaO Water and CO_2_ in the air. Furthermore, X-ray diffraction analysis revealed that the dried powder obtained from BiSCaO Water consisted primarily of calcium carbonate (CaCO_3_) (Figure 2).

### 2.2. Changes of pH in BiSCaO Water with Stirring in Open Air

**Figure 3:**
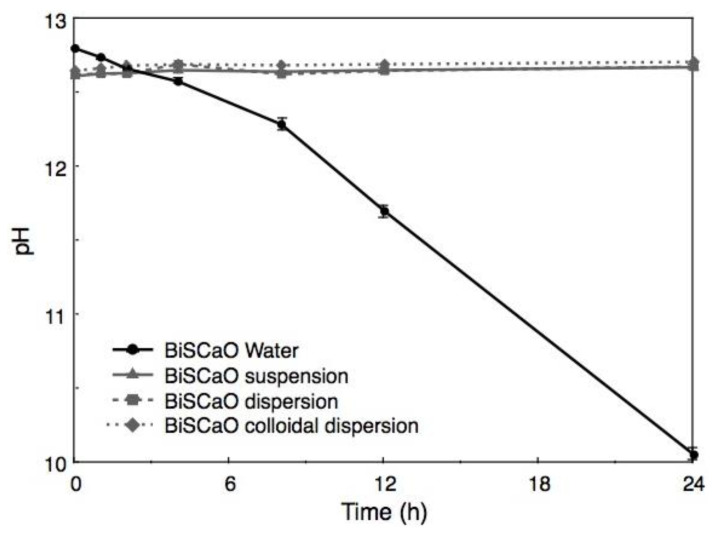
Changes of pH in BiSCaO Water during stirring in open air. When BiSCaO Water with strong alkalinity (pH 12.8) was exposed to air with stirring, the pH decreased to approximately 10 after 24 h. On the other hand, the pH in a BiSCaO suspension, dispersion, and colloidal dispersion did not change appreciably during 24 h of stirring in open air. Values are plotted as mean ± SD (*n* = 4).

When BiSCaO Water was exposed to air with stirring for 1 h, the liquid became cloudy. Furthermore, the pH decreased to 11.7 and 10.1 after 12 and 24 h, respectively, as shown in Figure 3. Following drying, the resulting white powder was insoluble; the supernatant of a suspension of this white powder had a pH of 9.6. On the other hand, the pH of a BiSCaO suspension (0.2 wt.% BiSCaO) [18,19,24], dispersion (0.2 wt.% BiSCaO + 0.12 wt.% Na_2_HPO_4_) [27], and colloidal dispersion (0.2 wt.% BiSCaO + 0.15 wt.% PP) [28] did not change during 24 h of stirring in open air. These results suggest that the insoluble powder was CaCO_3_ generated by an interaction between Ca^2+^ ions in BiSCaO Water and CO_2_ in the air, and that the BiSCaO suspension, dispersion, and colloidal dispersion contained insoluble CaO and/or Ca(OH)_2_ in the form of micro-/nano-particles or precipitates that provide hydroxyl ions (OH^–^) to maintain the alkaline pH.

### 2.3. Changes of pH in BiSCaO Water Sprayed on Surfaces of Materials and on Back of Hairless Rats

**Figure 4:**
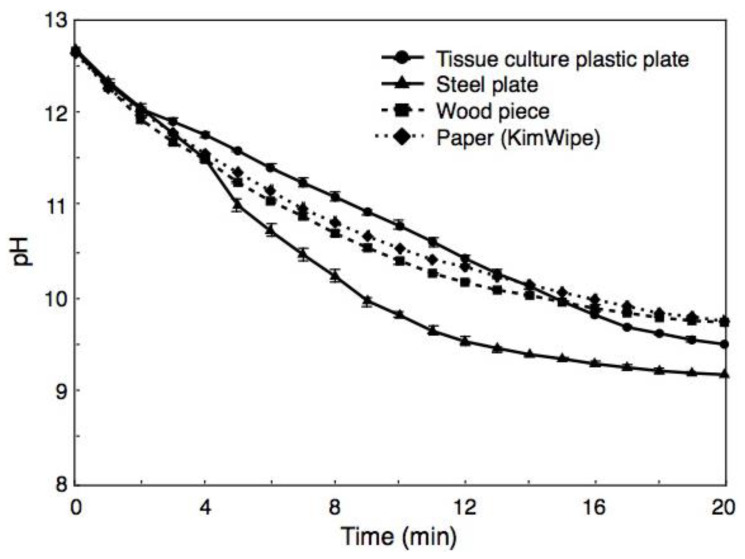
Changes of pH in BiSCaO Water sprayed on surfaces of materials. For all tested surfaces, the pH immediately after spraying was approximately 12.6, a value that was lower than the original pH of BiSCaO Water (i.e., pH 12.8). The pH on the surfaces continued to fall over the subsequent 20 min. Values are plotted as mean ± SD (*n* = 4).

BiSCaO Water was sprayed onto the surfaces of a plastic plate, a steel plate, a piece of wood, and tissue paper (KimWipe), and the pH of each wet surface was measured over the course of the next 20 min (Figure 4). For all tested surfaces, the pH immediately after spraying was 12.6 ± 0.02, a value that was lower than the original pH of BiSCaO Water (i.e., pH 12.8). The pH of the surfaces fell below 12 after 3 min, and continued falling to less than pH 11 and 10 after 10 and 17 min, respectively.

**Figure 5:**
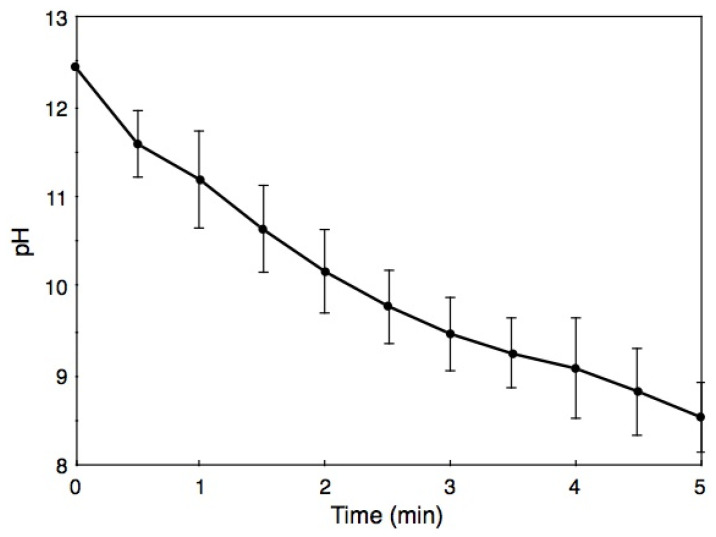
Changes of pH on back skin of hairless rats after spraying with BiSCaO Water. Values are plotted as mean ± SD (*n* = 4).

In a parallel experiment, BiSCaO Water (~1 mL) was sprayed onto the back skin of hairless rat, which then were rubbed lightly, and the pH of each wet skin was measured over the course of the following 5 min (Figure 5). The pH of the wet skin immediately after spraying was 12.4 ± 0.02, a value that was lower than the original pH of BiSCaO Water (i.e., pH 12.8). The pHs on the backs subsequently fell to pH 11.5 after 1 min, pH 9.5 after 3 min, and pH 8.5 after 5 min. We confirmed that none of the skin exhibited chapping or inflammation from use of BiSCaO Water.

### 2.4. Microbicidal Efficacy of BiSCaO Water, BiSCaO Water with 12-h Stirring, Suspension, Dispersion, and Colloidal Dispersion against Highly Contaminated Suspensions and Wood Pieces

**Figure 6:**
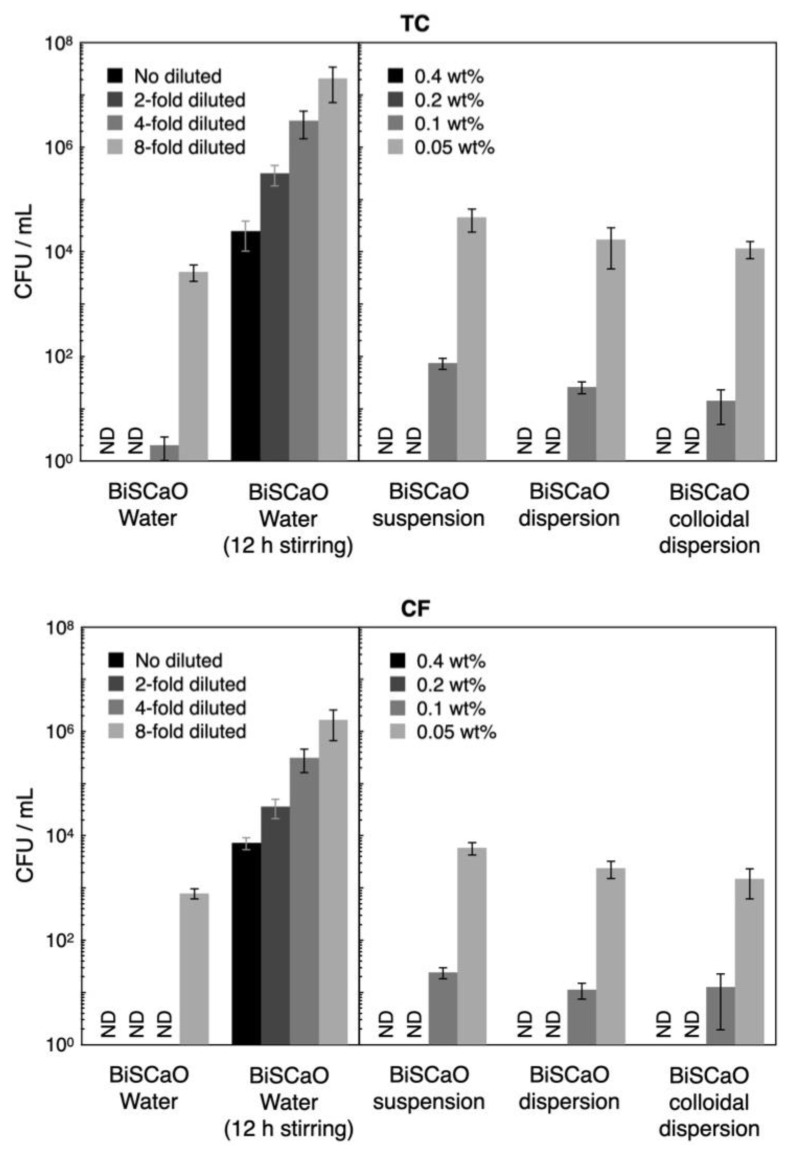
Microbicidal efficacy of test samples against contaminated suspension. The CFU/mL for total viable bacterial cells (TC) and coliform bacteria (CF) following treatment for 15 min with undiluted and two-fold diluted BiSCaO Water and 0.4 and 0.2 wt.% of BiSCaO suspension, dispersion, and colloidal dispersion could completely sterilize (<10 CFU/mL), while about 10,000 CFU/mL of both TC and CF with 12h-stirred BiSCaO Water remained viable even with the no diluted test sample. Plating and counting were performed as a set of four technical replicates (*n* = 4) and error bars represent means ± S.D. ND denotes not detected.

During incubation of bath tub water with 10% Dulbecco’s Modified Eagle’s Medium (DMEM) and 0.1 wt.% bovine serum albumin (BSA) at 37 °C for 24 h the TC and CF values increased from 80 ± 11 colony-forming unit (CFU)/mL and 32 ± 7 CFU/mL (respectively) to 9.8 × 10^7^ ± 3.1 × 10^7^ CFU/mL and 2.1 × 10^7^ ± 0.5 × 10^7^ CFU/mL, respectively. The CFU/mL for TC and CF following treatment for 15 min with undiluted (final two-fold diluted) and two-fold diluted (final four-fold diluted) BiSCaO Water and 0.4 and 0.2 wt.% (final 0.2 and 0.1 wt.%, respectively) of BiSCaO suspension, dispersion, and colloidal dispersion were below the detection limit (< 10 CFU/mL), whereas 10–100 and 1000–10,000 CFU/mL of both TC and CF remained viable following treatment with 0.1 wt.% (final 0.05 wt.%) and 0.05 wt.% (final 0.025 wt.%) of BiSCaO suspension, dispersion, and colloidal dispersion, respectively (Figure 6). In contrast, around 10,000 CFU/mL of TC and CF remained viable, even following treatment with undiluted (final 2-fold diluted) BiSCaO Water, when the reagent had been subjected to 12 h of stirring in ambient air.

**Figure 7:**
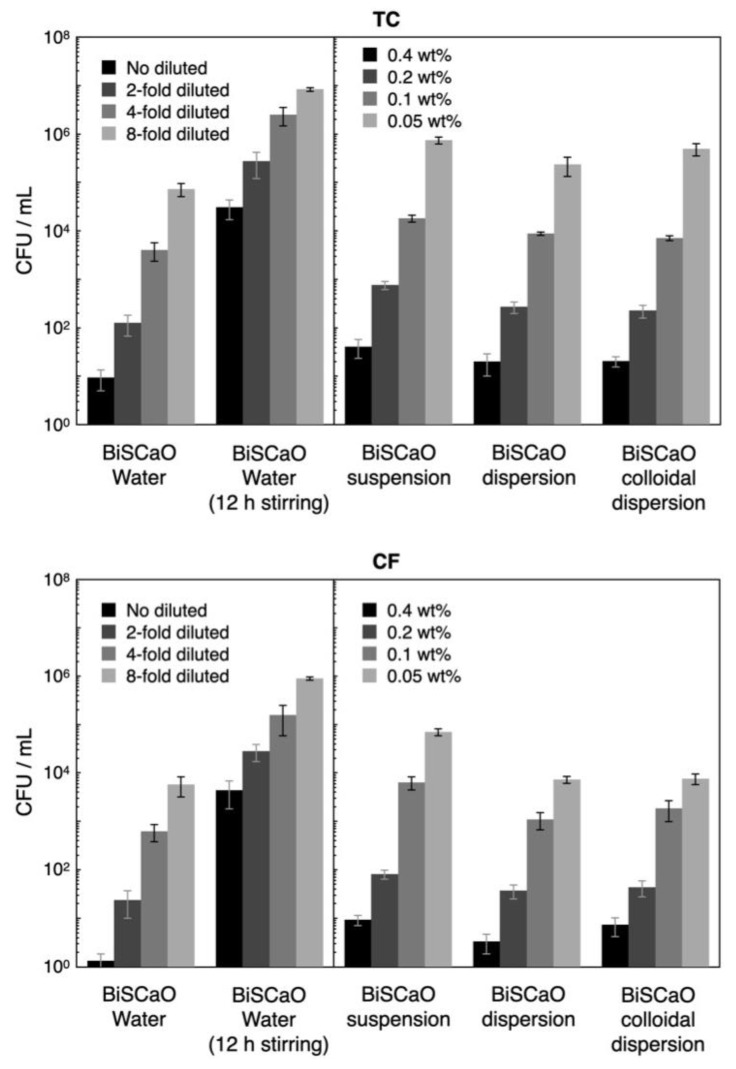
Fold diluted BiSCaO Water and 0.4 and 0.2 wt.% of BiSCaO suspension, dispersion, and colloidal dispersion showed disinfectant activities, but some TC and CF remained viable. Plating and counting were performed as a set of four technical replicates (*n* = 4) and error bars represent means ± S.D.

The CFU/mL for TC and CF released from the contaminated wood pieces was 2.5 × 10^7^ ± 0.6 × 10^7^ CFU/mL and 2.9 × 10^6^ ± 1.4 × 10^6^ CFU/mL, respectively. Although the CFU/mL for TC and CF following treatment with undiluted (final two-fold diluted) BiSCaO Water and 0.4 (final 0.2 wt.%) BiSCaO suspension, dispersion, and colloidal dispersion exhibited high disinfection activities (>5 log decreases in CFU/mL), a small part of TC (>1000 CFU/mL) and CF (>100 CFU/mL) remained viable (Figure 7). The CFU/mL for TC and CF following treatment with two-fold diluted (final four-fold diluted) BiSCaO Water 0.2 wt.% (final 0.1 wt.%,) BiSCaO suspension, dispersion, and colloidal dispersion exhibited >4 log decreases, while 100–1000 CFU/mL of TC and 10–100 CFU/mL of CF remained viable. In contrast, BiSCaO Water that had been subjected to 12-h stirring in ambient air exhibited weaker sterilization activity against both TC and CF, with TC (about 50,000 CFU/mL) and CF (about 7500 CFU/mL) remaining viable even following treatment even with undiluted (final two-fold diluted) BiSCaO Water with 12 h stirring (Figure 7). On the other hand, in BiSCaO suspension, dispersion, and colloidal dispersion the pH did not decrease at all with stirring for 12 h and their microbicidal activities against TC and CF also did not decrease (data not shown).

## 3. Discussion

Proper action plans for cleaning and disinfection play a critical role for minimizing the spread of infectious diseases such as COVID-19 during an outbreak. An effective action plan should outline what should be cleaned, the frequency of cleaning, the proper materials and techniques for disinfection, and the training of healthcare workers. The action plans using proper materials and techniques will support a facility’s pandemic response [1,2,3,30,31].

BiSCaO Water, which is a colorless and transparent disinfectant with a pH of 12.8, can be sprayed and dried on smooth metal or plastic surfaces, providing a white powder coating. Previously, SEM observation for the dried powder obtained from BiSCaO Water showed the presence of agglomerated microparticles (1–2 μm) [29]. In addition, cryo-SEM observation has indicated that aggregates (400–800 nm in size) of nanoparticles (100–200 nm) were contained in BiSCaO Water [29]. In the present work, elemental mapping of particles from BiSCaO Water revealed that the particles in the cloudy, air-exposed reagent, and the dried powder were composed of oxygen, carbon, and calcium (Figure 1). The dried white powder obtained from BiSCaO Water was insoluble in water, and the pH of the resulting supernatant was less than 10. Furthermore, X-ray diffraction analysis confirmed that the dried powder recovered from BiSCaO Water consisted primarily of CaCO_3_ (Figure 2).

When BiSCaO Water was exposed to air with stirring, the liquid became cloudy after 1 h. Furthermore, the pH decreased to approximately 11.7 and 10.1 after 12 and 24 h of stirring, respectively (Figure 3). In contrast, the pH of a BiSCaO suspension, dispersion, and colloidal dispersion did not decrease when treated similarly. These results suggest that the insoluble powder from BiSCaO Water is CaCO_3_, which presumably is generated by the interaction between Ca^2+^ ions in BiSCaO Water and CO_2_ in the air, and that a BiSCaO suspension, dispersion, and colloidal dispersion contained insoluble CaO and/or Ca(OH)_2_ in the form of micro-/nano-particles or precipitates that provide hydroxyl ions (OH^–^) to maintain the elevated pH. BiSCaO Water after 12h stirring, which had decreased pH and contained CaCO_3_, exhibited much less microbicidal activities than did fresh BiSCaO Water (Figure 6 and Figure 7). 

We have previously reported that BiSCaO Water was a potent reagent with excellent deodorization and disinfection activities against pathogenic bacteria and viruses including both enveloped and nonenveloped viruses due to the strong alkaline [29]. However, there has been serious concern about the safety of BiSCaO Water for use on living tissues, given that this reagent has a pH exceeding 12.7. This study showed that BiSCaO Water, when BiSCaO Water was sprayed onto a variety of surfaces (plastic plate, steel plate, wood, and paper), gave an initial pH of 12.6 ± 0.02, a value lower than the original value (pH 12.8) for BiSCaO Water, with the pH on the surfaces subsequently and rapidly decreasing to below 12, 11, and 10 after 3, 10, and 20 min, respectively (Figure 4). Thus, the high pH of this fresh reagent rapidly decreases on surfaces and skin due to the generation of CaCO_3_ that lack obstacles following the interaction between Ca^2+^ ions in BiSCaO Water and CO_2_ in the air. In fact, CaCO_3_ powder is used as a gastric antacid [32], an anti-erosive agent [33], and a plant fertilizer [34]. We infer that the ingestion of a small amount of BiSCaO Water would not cause problem if swallowed or inhaled, given that CaCO_3_ powder will be converted to a safe and soluble compound (calcium bicarbonate (Ca(HCO_3_)_2_)) upon further interaction between CaCO_3_ and CO_2_. These results in this study suggest that spraying BiSCaO Water onto the surfaces of various materials is safe cleaning procedure in spite of its strong initial alkalinity in addition to the strong disinfectant activity.

Furthermore, we have already reported that cleansing *P. aeruginosa*-infected wounds with transparent supernatant liquids of 0.2 wt.% BiSCaO suspension for 3 days enhanced removal of the bioburden and wound repair without any complications such as acute inflammation, abscess formation, or seroma accumulation [24]. Similarly, the pH of BiSCaO Water sprayed onto hairless rat skin fell to pH 11.5 after 1 min and pH 9.5 after 3 min of application (Figure 5) without apparent harmful effects such as rough skin. Thus, BiSCaO Water appears to be friendly to skin. Nevertheless, it is necessary to perform the safety study for BiSCaO Water to apply onto human such as hand sterilization and mouth cleaning with official ethics committee approval on use of human subjects.

The World Health Organization (WHO) recommends “to ensure that environmental cleaning and disinfection procedures are followed consistently and correctly. Thoroughly cleaning environmental surfaces with water and detergent and applying commonly used hospital-level disinfectants (such as sodium hypochlorite) are effective and sufficient procedures” [1]. Although some antiseptics/disinfectants, including ethanol and NaClO, exhibit significant activity against SARS-CoV-2 by damaging the virus envelope, they are cytotoxic to cellular and organic components and require high concentrations for sufficient activity [4,5,6]. Furthermore, the presence of organic materials significantly diminishes the activity of chlorine-derived compounds [6,7]. Therefore, novel antiseptics/disinfectants without harmful side effects or environmental disruption are still desired for environmental hygiene and public health. We anticipate that BiSCaO Water with attractive characteristics, including its apparent safety and virucidal activity against enveloped viruses, may be a trump card against respiratory viruses such as SARS-CoV-2, even though further studies are required to validate the activity of BiSCaO Water against critical pathogens, including the coronavirus. 

## 4. Materials and Methods 

### 4.1. BiSCaO Powder and Chemicals.

Scallop shell powder that had been heated at 1450 °C for 4 h (average BiSCaO particle diameter of 6 μm) was purchased from Plus Lab Corp., Kanagawa, Japan. According to the manufacturer, the CaO concentration in all BiSCaO preparations exceeded 99%. Na_2_HPO_4_ (197-02865) and Na-polyPO_4_ (PP) (694-05935) were purchased from FUJI FILM Wako Pure Chemical Corp., Osaka, Japan.

### 4.2. BiSCaO Suspensions, BiSCaO Dispersion with Na_2_HPO_4_, BiSCaO Colloidal Dispersion with PP, and BiSCaO Water

BiSCaO was added to pure water followed by rotary mixing to generate BiSCaO suspensions, to which a solution of 60% Na_2_HPO_4_ or 75% PP compared with BiSCaO was added to prepare BiSCaO dispersions or BiSCaO colloidal dispersions, respectively [27,28].

BiSCaO Water was purchased from Plus Lab Corp. According the manufacturer, BiSCaO Water was prepared by adding 100 g of BiSCaO to 1100 mL chilled (<10 °C) clean water; the combination then was gently mixed and allowed to stand for 30 min. The resulting supernatant (1000 mL) was decanted to a 100 L water tank. Another chilled 1000 mL of clean water was gently added to the remaining BiSCaO precipitate; again the combination was gently mixed and allowed to stand for 30 min, and the supernatant was decanted into the collection tank. This process was repeated for a total of a hundred times. The resulting BiSCaO Water (total 100 L) was obtained commercially from Plus Lab Corp.

### 4.3. SEM Images of BiSCaO Water and X-ray Diffractograms of the Dried Powder

SEM observation and EDX elemental mapping of freeze-dried powder of BiSCaO Water were performed by outsourcing to JEOL, Ltd., Tokyo, Japan. The observations were conducted using a JEOL JSM-7200F microscope (JEOL Ltd., Tokyo, Japan) equipped with a JED-2300 EDX analysis system at an accelerating voltage of 10 kV at room temperature.

The content of dried powder derived from BiSCaO Water was determined using an X-ray diffractometer system (Phillips X’Pert-PRO; Phillips Japan, Ltd., Japan). This analysis was performed at the Kanagawa Institute of Industrial Science and Technology.

### 4.4. Changes of pH in BiSCaO Water with Stirring in Open Air

Each aliquot (200 mL) of BiSCaO Water, BiSCaO suspension (0.2 wt.% BiSCaO) [18,19], dispersion (0.2 wt.% BiSCaO + 0.12 wt.% Na_2_HPO_4_) [27], or colloidal dispersion (0.2 wt.% BiSCaO + 0.15 wt.% PP)) [28] was dispensed into a 500-mL beaker and actively stirring using Digital Stirrer/Hotplate (Corning Japan Inc., Tokyo, Japan) with stirring bar (5 mm in diameter × 50 mm) (As One Corp. Osaka, Japan) for 24 h at room temperature in ambient air. The pH of each BiSCaO formulation was measured (using a bench pH meter; F-52, HORIBA, Ltd., Kyoto, Japan) at 0, 1, 2, 4, 8, 12, and 24 h during the process.

### 4.5. Changes of pH in BiSCaO Water Sprayed on Surfaces of Materials and on Back of Hairless Rats

When an aliquot (approximately 2 mL) of BiSCaO Water was sprayed onto a defined surface (about 5 cm × 5 cm) of a culture plate and a steel plate, generations of large and small droplets (diameter: 1–3 mm, thickness: 0.5–2 mm) were observed. The pH of large droplet was measured every 1 min during 20 min using diagonally placed Micro Tough electrode (9618S-10D; HORIBA, Ltd.) attached to a bench pH meter (LAQUA pH meter, F-74, HORIBA, Ltd.). Similarly, approximately 1 mL of BiSCaO Water were sprayed onto the back skin of hairless rats (male, 300–350 g) from Japan SLC Inc., Shizuoka, Japan and maintained under appropriate condition (i.e., 26 °C and 55% humidity). In the experiment, the rats were placed under general anesthesia by intraperitoneal injection of pentobarbital sodium (Dainippon Sumitomo Parma Co., Ltd., Osaka, Japan) [19,24,25]. The pH of BiSCaO Water on the back skin of the rats was measured every 1 min during 5 min using diagonally placed Micro Tough electrode attached to the pH bench meter. All animal experiments were approved by the National Defense Medical College, Tokorozawa, Saitama, Japan, and carried out following the relevant guidelines for animal experimentation (Approval number, 17084, 18/2/2019). On the other hand, the spraying approximately 2 mL of BiSCaO Water onto surface (about 5 cm × 5 cm) of wooden pieces and paper sheets (KimWipe, Nippon Paper Crecia, Co., Ltd., Tokyo, Japan) were permeated into them and did not generate droplets. However, the pH of their wet surface could be measured using diagonally placed Micro Tough electrode attached to the pH bench meter for 20 min.

### 4.6. Microbicidal Activity of BiSCaO Water, BiSCaO Water with 12-h Stirring, Suspension, Dispersion, and Colloidal Dispersion against Highly Contaminated Suspensions and Wood Pieces

We investigated the microbicidal efficacy of variously dilutions of BiSCaO Water and BiSCaO Water with 12-h stirring, as well as various concentrations of BiSCaO suspension, dispersion, and colloidal dispersion. These reagents were tested against a contaminated suspension comprising normal bacterial flora (CF and TC), which was prepared by incubating the leftover bath water with 10% DMEM and 0.1wt% BSA at 37°C for 24 h [18,19,26]. Equal volumes of each test sample and the contaminated suspension were mixed well and incubated at room temperature for 15 min, and then the density of CFU/mL per sample was determined. For the disinfection assay of contaminated wood pieces, a wood piece was added to 10 mL of each disinfectant, rinsed gently for 15 min, and then TC and CF were released from the contaminated wood piece in 10 mL of clean sterile water by vigorous vortexing for 2 min. To evaluate CFU, 1 mL of each mixture was gently added to individual Petri dishes with pre-aliquoted portions of simple and easy dry medium for TC or CF (Nissui Pharmaceutical Co., Ltd., Tokyo, Japan) [18,19,27,28], followed by incubation for 24 h in a 37 °C incubator (A1201; IKUTA Sangyo Co., Ltd., Ueda, Nagano, Japan). Plating and counting experiments were conducted as a set of 4 technical replicates (*n* = 4). 

## 5. Conclusions

BiSCaO Water is colorless and transparent and has a pH of 12.8. This reagent has higher deodorization and disinfection activities than do other microbicidal reagents such as ethanol, NaClO, and povidone iodine [18,19]. However, concerns have been raised concerning the safety of BiSCaO Water when applied to the living body, given the strong alkalinity of this reagent. The present study showed that the high initial pH of BiSCaO Water following application to various surfaces and skin of hairless rat, rapidly decreases for areas that lack obstacles with the generation of CaCO_3_ as a result of the interaction between Ca^2+^ in BiSCaO Water and CO_2_ in the air. Thus, the characteristics of BiSCaO Water, including both its safety and microbicidal activity, may be valuable for limiting the spread of various pathogenic microbes and viruses.
